# ASSESSING THE PSYCHOMETRIC PROPERTIES OF THE GERIATRIC SUICIDE IDEATION SCALE IN MIDDLE-AGED AND OLDER MEN

**DOI:** 10.1093/geroni/igad104.0941

**Published:** 2023-12-21

**Authors:** Marnin Heisel, Gordon Flett

**Affiliations:** University of Western Ontario, London, Ontario, Canada; York University, Toronto, Ontario, Canada

## Abstract

Middle-aged and older men have high rates of suicide, necessitating risk detection. We developed the Geriatric Suicide Ideation Scale (GSIS; Heisel & Flett, 2006) as an age-specific, multidimensional suicide risk assessment tool. The GSIS has shown strong psychometric properties in clinical, community, and residential samples (see Heisel & Flett, 2016), yet research has lagged investigating its utility with middle-aged and older men. The purpose of the present study was thus to assess the psychometric properties of the GSIS administered to 82 men, 55 years and older (M=63.3, SD=4.6 years), who participated in a meaning-centered psychological intervention group for those concerned about or struggling with the transition to retirement (Heisel et al., 2020). Psychometric analyses included investigation of participant response characteristics, internal consistency, and construct validity. Findings demonstrated acceptable internal consistency for GSIS totals (α =.88) and for its Suicide Ideation, Death Ideation, Loss of Personal and Social Worth, and Perceived Meaning in Life subscales (α =.62-.81). Positive associations between the GSIS and negative psychological factors (depression, anxiety, hopelessness, loneliness, perceived lack of mattering to others, and history of suicidal behavior; r =.30 to .51) and negative associations with positive factors (life satisfaction, psychological well-being, perceived support, and meaning in life; r = -.21 to -.51) supported its construct validity. These and other findings will be discussed in the broader context of upstream population level approaches to suicide risk detection and prevention. (Note: this abstract was accepted for the 2022 GSA meeting, but we had to withdraw due to family loss)

